# Altered leaf functional traits by nitrogen addition in a nutrient-poor pine plantation: A consequence of decreased phosphorus availability

**DOI:** 10.1038/s41598-017-07170-3

**Published:** 2017-08-07

**Authors:** Lin-Lin Zheng, Qiong Zhao, Zhan-Yuan Yu, Shan-Yu Zhao, De-Hui Zeng

**Affiliations:** 1 Key Laboratory of Forest Ecology and Management, Institute of Applied Ecology, Chinese Academy of Sciences, Shenyang, 110016 P.R. China; 2 Daqinggou Ecological Station, Institute of Applied Ecology, Chinese Academy of Sciences, Shenyang, 110016 P.R. China; 3 University of Chinese Academy of Sciences, Beijing, 100049 P.R. China

## Abstract

This study aimed to determine how specific leaf area (SLA) and leaf dry matter content (LDMC) respond to N addition and understory vegetation removal in a 13-year-old Mongolian pine (*Pinus sylvestris* var. *mongolica*) plantation. Traits (SLA, LDMC, individual needle dry weight, N and P concentrations) of different-aged needles and their crown-average values were measured, and their relationships with soil N and P availability were examined. N addition and understory removal reduced soil Olsen-P by 15–91%. At the crown level, N addition significantly reduced foliar P concentration (by 19%) and SLA (by 8%), and elevated N concentration (by 31%), LDMC (by 10%) and individual leaf dry weight (by 14%); understory removal did not have a significant effect on all leaf traits. At the needle age level, traits of the previous year’s needles responded more strongly to N addition and understory removal than the traits of current-year needles, particularly SLA and N concentration. SLA and LDMC correlated more closely with soil Olsen-P than with soil inorganic N, and LDMC correlated more closely with soil Olsen-P than SLA did. These results indicate that aggravated P limitation resulting from N addition and understory removal could constrain Mongolian pine growth through their effects on the leaf traits.

## Introduction

Enhanced nitrogen (N) deposition and the elimination of understory vegetation are two of the main drivers that affect soil nutrient availability in managed forests, and the altered nutrient availability has great potential to change the ecosystem structure and functioning. Human activities have more than doubled the rate of N input into terrestrial ecosystems worldwide, with this rate still increasing^1^. Anthropogenic N addition can greatly affect ecosystem structure and functioning, such as species composition, productivity and stability, not only by directly increasing soil N availability and altering the N cycle, but also by altering the availability of other nutrient elements, in particular phosphorus (P)^1–3^. Increased N supply can profoundly affect P availability by altering soil phosphatase activity, soil pH and plant P uptake^2–4^. N addition has been extensively studied worldwide, but its consequences on ecosystem processes are highly variable, and the underlying mechanisms are still not well understood^2^. Soil nutrient availability in managed forests can also be altered by management practices, such as the removal of understory vegetation. Traditionally, understory vegetation is removed in managed forests to reduce resource competition between trees and the understory community^5^. Increased soil available N in forest plantations by eliminating understory vegetation has been widely observed^6–8^. However, recent studies suggest that understory vegetation plays a positive role in maintaining the long-term structure and function of managed forests^9, 10^. Understory plant community can increase forest nutrient retention, as well as mediate the litter decomposition of overstory trees, due to its higher biomass turnover rate and more easily decomposable litter relative to the overstory trees^11–13^. More fieldwork is still needed to gain a better understanding of how the N deposition and understory removal affect ecosystem functioning.

A set of easily measured leaf traits has been identified as useful predictors of ecosystem functioning^14, 15^. Specific leaf area (SLA, defined as leaf area per unit dry mass) and leaf dry matter content (LDMC, defined as leaf dry mass per unit water-saturated fresh leaf mass), are key indicators of plant growth and strategy for resource acquisition and conservation, due to their tight link to relative growth rate, leaf net photosynthetic rate and leaf life span^16–18^. SLA reflects the ability of leaves to capture light, and consists of two components: leaf thickness and density (dry weight per unit volume), and the density is algebraically equivalent to LDMC. The merit of SLA makes it serve as an important input parameter to many large-scale ecosystem models^19^. SLA and LDMC not only vary among plant species but also react sensitively to variations in resource availability, e.g., light, humidity and nutrients^18, 20^. Considering that climate change and human disturbances have substantially altered nutrient availability and cycles^1, 21^, understanding how the variation in nutrient availability affects SLA and LDMC is urgent for predicting the dynamics of ecosystem functioning in a changing world.

The influence of soil nutrient availability on SLA is complex and largely uncertain. For trees, many studies conducted along natural nutrient gradients revealed positive impacts of increased soil nutrient availability on SLA^22–24^, but the negative or neutral impacts were often obtained from fertilization experiments^25–27^. Several factors have been considered as possible causes for the inconsistent results between studies, such as extent of nutrient limitation, covariation in light availability and tree species^18, 23^. Additionally, other factors could be largely responsible for the uncertainty in the relationship between soil nutrient availability and SLA, such as interactions between the availability of different nutrient elements and methodological problems of measuring SLA and leaf collection. Interactions among the availabilities of N, P and other elements can confuse the impacts of soil nutrient availability on SLA. Increases in the supply of one nutrient element can alter the supply of other nutrients, which may cause the nutrient imbalance in plants^2, 28^. However, most previous studies focused on the variation in a single nutrient, usually N, while the covariation in the supply of P and other elements has seldom been taken into account, even though variation in N supply can substantially affect the availability of P and other nutrient elements^2–4, 29^. Some studies suggest that LDMC is a better predicator of soil nutrient availability than SLA, as LDMC is more easily and accurately measured and less dependent on leaf thickness than SLA^17, 30, 31^. In addition, most previous studies on the leaf traits of *Pinus* only measured the current-year needles without considering the previous year’s needles, despite old needles possibly being more sensitive to variations in soil nutrient availability than new needles due to the transfer of nutrients from old to new leaves^32^.

The overall goal of this study was to determine how variations in soil N and P availability, induced by N addition and understory vegetation removal, affect foliar chemistry and morphology in a nutrient-poor Mongolian pine plantation. To achieve our goal, we examined the morphological traits and nutrient concentrations of different-aged needles and soil physiochemical properties and analyzed their correlations in a pure Mongolian pine (*Pinus sylvestris* var. *mongolica*) plantation that has been subjected to five years of N addition and understory removal. We expected that: (1) N addition would elevate N availability, but decrease soil P availability by depressing soil microbial activity and acidifying the soil^3, 33^. (2) The removal of understory vegetation would increase soil available N and P concentrations, as it will reduce plant nutrient uptake. (3) SLA and LDMC would be more strongly correlated with soil available P than with available N, as N addition is expected to reduce soil P availability and thus intensify P limitation. (4) The previous year’s needles would be more sensitive to variations in nutrient availability than current-year needles. Given that Mongolian pine is one of the most widespread tree species planted at infertile sites with low N and P availability in northern China^34, 35^, the results of the present study can provide useful information for simulating and predicting the growth and adaptation responses of pine stands to variations in nutrient availability induced by climate change and artificial disturbances.

## Results

### Soil physiochemical properties

N addition significantly elevated total N concentration and reduced soil pH, while understory removal and its interaction with N addition did not affect these variables (Supplementary Table 1). Soil Olsen-P concentration was significantly affected by N addition and its interaction with understory removal (Supplementary Table 1) as it was 15%, 91% and 39% lower in the U− (understory removal), N+ (addition of 10 g N m^−2^ year^−1^) and N + U− (combination of N addition and understory removal) plots than in the control plots (the control without any disturbance, Table 1), respectively. Soil NO_3_-N and NH_4_^+^-N concentrations were significantly affected by N addition and its interaction with understory removal, but not by understory removal alone (Supplementary Table 1). Soils in the N+ and N + U− plots had 2.8 and 4.4 times higher NO_3_-N and 5.4 and 11.9 times higher NH_4_^+^-N concentration than soils in the control plots (Table 1), respectively. N addition and understory removal both significantly reduced microbial biomass C (MBC) concentration, by an average of 26% relative to the control (Table 1 and Supplementary Table 1). There were no significant effects of any treatment on soil water content and concentrations of total P and soil organic C (SOC).

**Table 1:** Soil properties as influenced by N addition and understory removal in a Mongolian pine plantation.

Treatment	pH	Water content (%)	NO_3_^-^N (mg kg^−1^)	NH_4_^+^-N (mg kg^−1^)	MBC (mg kg^−1^)	Olsen-P (mg kg^−1^)	SOC (g kg^−1^)	Total N (g kg^−1^)	Total P (g kg^−1^)
**Control**	6.48 ± 0.18a	7.58 ± 0.67a	2.02 ± 0.38c	1.05 ± 0.06c	208.26 ± 20.89a	2.16 ± 0.20a	3.73 ± 0.49a	0.35 ± 0.05ab	0.1 ± 0.01a
**U−**	6.22 ± 0.05a	7.07 ± 0.48a	1.9 ± 0.32c	1.24 ± 0.26c	155.21 ± 9.86b	1.88 ± 0.13b	3.63 ± 0.43a	0.29 ± 0.12b	0.09 ± 0.01a
**N+**	5.51 ± 0.28b	8.4 ± 1.05a	7.59 ± 0.36b	6.68 ± 0.41b	162.83 ± 4.78b	1.13 ± 0.02d	4.89 ± 0.79a	0.47 ± 0.10a	0.11 ± 0.01a
**N + U−**	5.21 ± 0.2b	7.38 ± 0.53a	10.91 ± 1.3a	13.54 ± 0.72a	147.05 ± 9.17b	1.55 ± 0.14c	4.21 ± 0.41a	0.4 ± 0.07ab	0.09 ± 0.01a

Values are means ± standard error, *n* = 4. Different letters within each column indicate significant differences (*P* < 0.05), according to the LSD post-hoc test following one-way ANOVA.

*Control* control plots, *U*− understory removal, *N* + N addition, *N* + *U*− N addition with understory removal. *MBC* microbial biomass C, *SOC* soil organic C.

**Figure 1:**
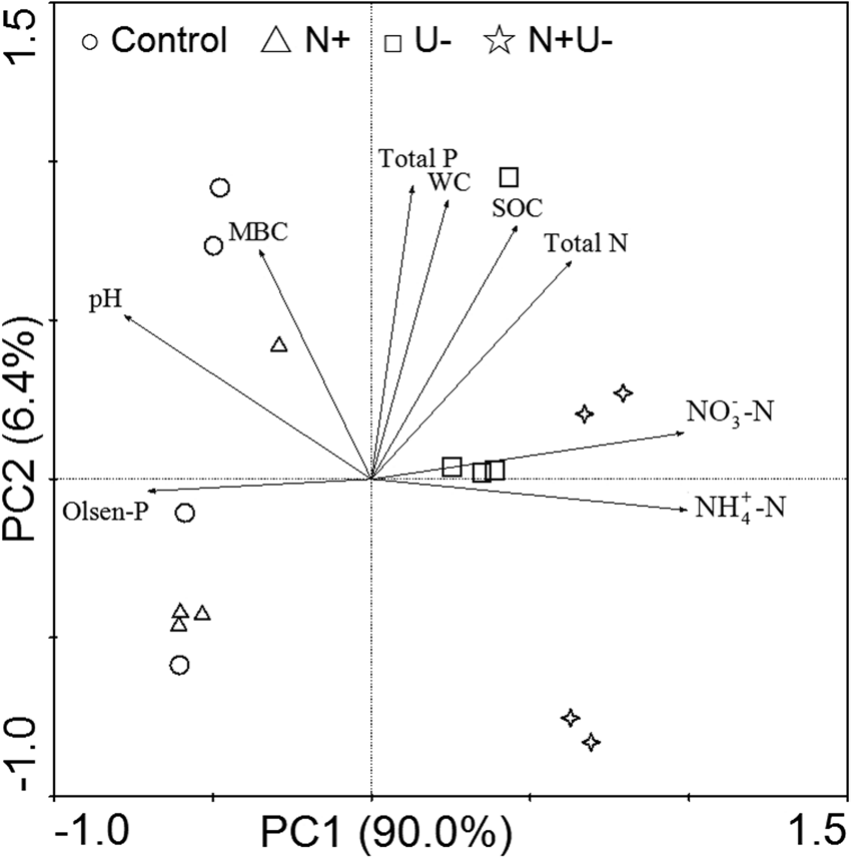
Ordination diagram with variables and samples from principal component analysis (PCA) of soil variables in a Mongolian pine plantation. Each symbol represents a sample. Lines with arrow represent soil variables, and arrow points in the direction of steepest increase of values. The smaller angle between two variable arrows indicates stronger correlation, as the cosine of the angle between variable arrows equals their correlation coefficients. N+: N addition; U−: Understory removal.

Pearson’s correlation analysis showed that soil NO_3_-N and NH_4_^+^-N concentrations were correlated negatively with Olsen-P (*r* = −0.61, *p* = 0.013, and *r* = −0.56, *p* = 0.023) and pH (*r* = −0.67, *p* = 0.005, and *r* = −0.80, *p* = 0.000), but not with other soil variables. Soil Olsen-P and MBC were correlated positively with pH (*r* = 0.67, *p* = 0.004, and *r* = 0.57, *p* = 0.02). The biplot from PCA clearly visualized the above correlations among soil properties (Fig. 1). The first principal component (PC1) explained 90.0% of the total variance and was mainly associated with NO_3_-N, NH_4_^+^-N, Olsen-P and pH. The second principal component (PC2) explained only 6.4% of the total variance and was primarily related to soil water content, MBC, SOC, total N and total P (Fig. 1).

### Needle nutrition and morphological traits

**Figure 2:**
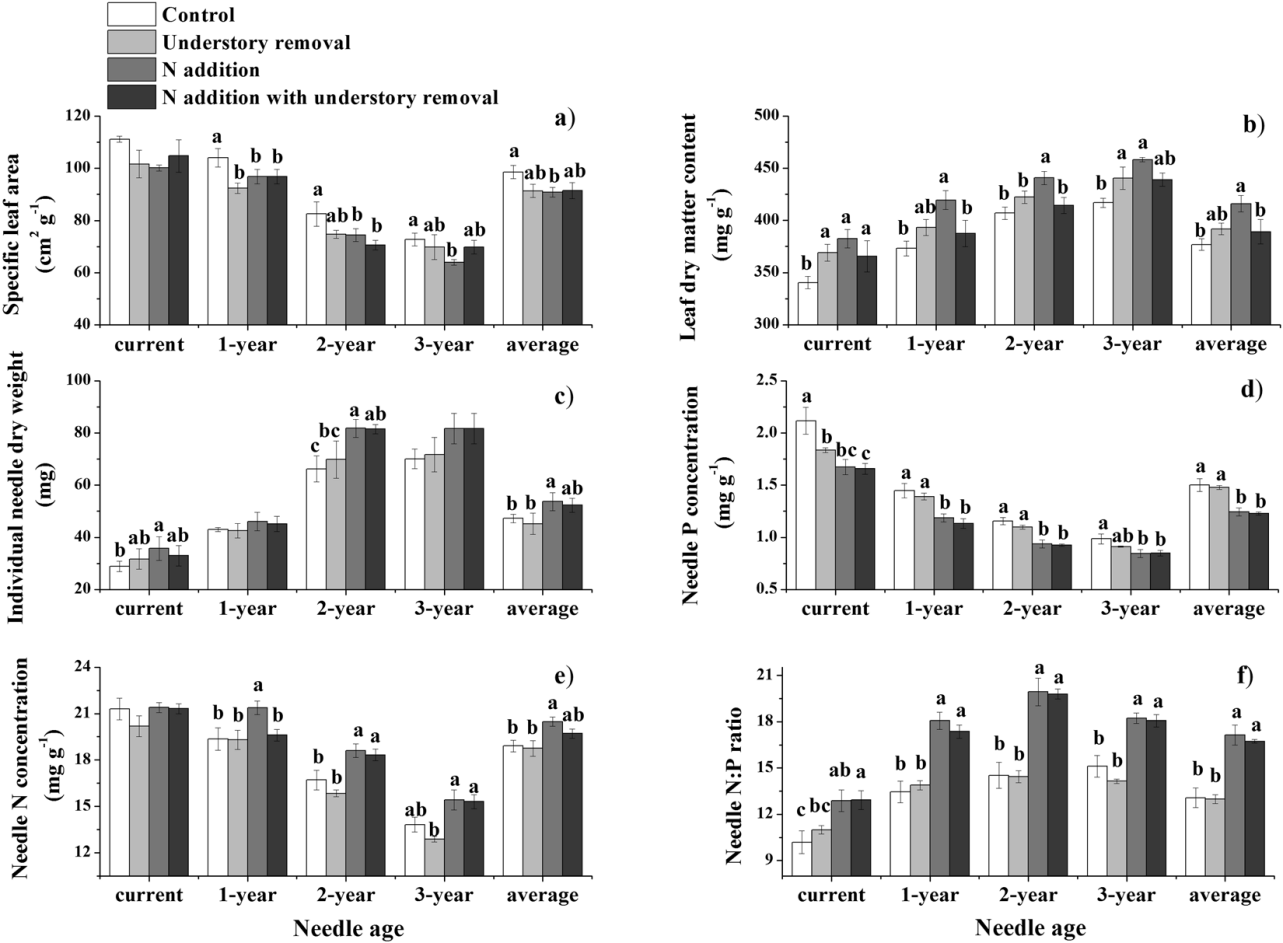
Changes in morphological and chemical traits of different-aged needles and their crown averages in response to N addition and understory removal in a Mongolian pine plantation. Values are means ± standard error, n = 4. Different letters within each age class indicate significant differences (*p* < 0.05), according to the LSD post-hoc test following ANOVA.

Based on our tests for the treatment effects on the mean values of needle traits at the crown level, we found that all needle traits were significantly affected by N addition, but not by understory removal (Supplementary Table 1). Trees in the N+ plots had significantly lower SLA (8%), and higher LDMC (10%) and individual leaf dry weight (14%) than trees in the control plots (Fig. 2). Needle N:P ratio and N concentration were significantly higher (4–31%) and needle P concentration was significantly lower (19–22%) in the N+ and N + U− plots than in the control plots, respectively (Fig. 2).

When we tested for the effects of treatment on needle traits separately for different-aged needles, we found that our experimental treatments generally had similar effects on needle traits for all age classes, but the magnitude of these effects largely depended on needle age, with the greater effects on 2- and 3-year-old needles. N addition and understory removal reduced SLA by 8–17%, and the reduction was insignificant for current-year needles (Fig. 2a). LDMC was significantly higher (8–12%) in the N+ plots than in the control plots for needles of all age classes and was significantly affected by the U− and N + U− treatments only for current-year and 3-year-old needles (by approximately 5%, Fig. 2b). The treatment effects on needle dry weight were generally greatest for 2-year-old needles (Fig. 2c). N addition significantly reduced P concentration (15–28%) and increased the N:P ratio (21–37%) in needles of all age classes, and generally increased N concentration (3–11%) in all previous year’s needles but not in the current-year needles (Fig. 2d,e and f). Understory removal had almost no significant effects on needle N and P concentrations and the N:P ratio for needles of all age classes.

Needle age had strong direct effects on most of the needle traits. The individual dry weight significantly increased by 1.4 times, LDMC increased by 20%, and N:P ratios increased by 37% with increasing needle age; SLA decreased by 34%, and the concentration of P and N decreased by 51% and 32%, respectively, with increasing needle age (Supplementary Table 2).

### Correlations between needle traits and soil properties

**Figure 3:**
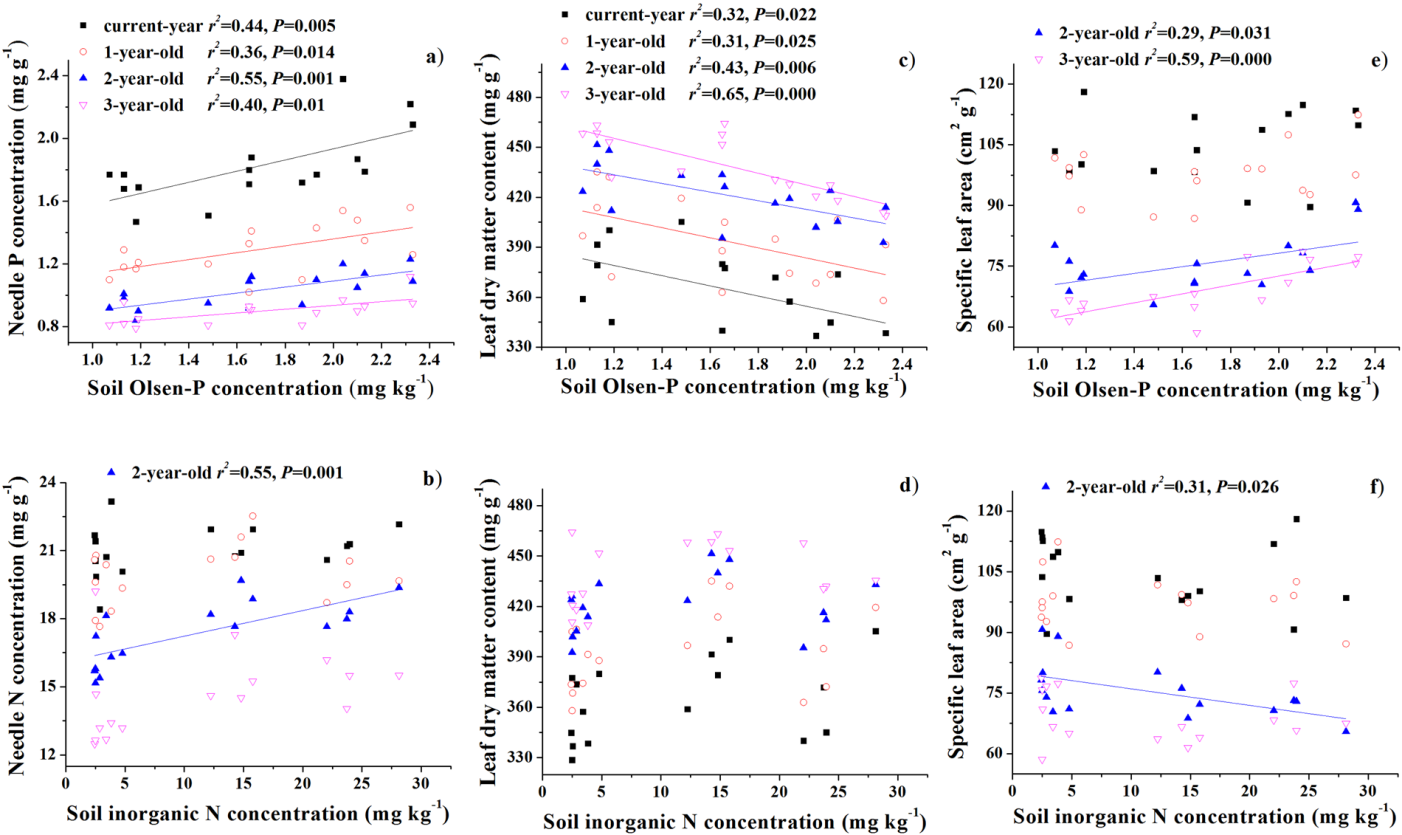
Relationships between needle traits and soil properties (inorganic N and Olsen-P) in a Mongolian pine plantation. The data were fitted by linear regression, and fitted regression lines denote significant regressions (*p* < 0.05).

To reveal how the variation in soil nutrient availability affects needle nutritional and morphological traits, we conducted the Pearson’s correlation analyses between soil nutrient availability (indicated by soil inorganic N and Olsen-P) and needle traits at the crown and needle age levels. At the crown level, and for needles of all age classes, needle P concentration was significantly correlated with soil Olsen-P concentration (*r* = 0.60–0.74, *p* < 0.02, Table 2 and Fig. 3a), and N:P ratio was positively correlated with soil inorganic N (*r* = 0.72–0.84) and negatively with Olsen-P (*r* = −0.63– − 0.79, *p* < 0.01, Table 2). In contrast, needle N concentration was significantly correlated with soil inorganic N concentration only for 2-year-old needles (*r* = 0.74, *p* = 0.001) and for the mean value at the crown level (*r* = 0.53, *p* = 0.035, Fig. 3b).

**Table 2:** Pearson’s correlation coefficients (*P* values in the parenthesis) between crown average values of needle traits and soil available nutrients.

	DW	SLA	LDMC	LNC	LPC	N:P ratio
**Inorganic N**	**0.538 (0.031)**	−0.399 (0.126)	0.314 (0.236)	**0.528 (0.035)**	**−0.833 (0.000)**	**0.824 (0.000)**
**Olsen-P**	−0.455 (0.077)	0.379 (0.148)	**−0.617 (0.011)**	**−0.744 (0.001)**	**0.700 (0.003)**	**−0.788 (0.000)**

*DW* individual needle dry weight, *SLA* specific leaf area, *LDMC* leaf dry matter content, *LNC* leaf N concentration, *LPC* leaf P concentration.

At the crown level, LDMC was positively correlated with soil Olsen-P, and not correlated with soil inorganic N; SLA was not correlated with either Olsen-P or inorganic N. While individual needle dry weight was significantly correlated with inorganic N (Table 2). For specific age classes, LDMC was still positively correlated with soil Olsen-P, but not with inorganic N for needles of all age classes (Fig. 3c and d); SLA was positively correlated with soil Olsen-P for 2- and 3-year-old needles, and negatively with soil inorganic N for 2-year-old needles (Fig. 3e and f).

## Discussion

Soil Olsen-P was reduced by both N addition and understory removal in the Mongolian pine plantation, with a stronger effect of N addition (Table 1). This result supports our first expectation that N addition would reduce soil available P, but contradicts our second expectation that the removal of understory vegetation would increase soil available N and P. In the present study, understory removal slightly reduced soil Olsen-P concentration and did influence inorganic N, the mechanisms of which are difficult to explain, as we did not fully study the N and P transformation processes. Removal of understory vegetation can not only directly increase available soil nutrients by decreasing nutrient uptake but can also indirectly influence nutrient cycling processes via many ways, such as its impacts on soil microorganisms, temperature, moisture, and rhizodeposition^7, 8, 13^. Understory removal was found to decrease soil microbial biomass and activity, as well as increase nitrate leaching and N_2_O flux in forest plantations^8, 13, 36^. Increased and unchanged available N from understory removal has been reported previously^6, 7, 37^. N addition can affect P availability via altering the soil pH and phosphatase activity, and via promoting P uptake^2–4^. Soil pH is a primary factor affecting soil P availability. When soil pH ranges from 4 to 6.5, soil available P decreases with the reduction in pH, because more phosphate is adsorbed by or precipitated with Fe^3+^, Al^3+^, and Mn^2+^ ions that are released with soil acidification^37^. Soil acidification is one of the main consequences of N deposition for soils with low buffer capacity^1^; this is the case in the present study, as N addition reduced soil pH by 1 unit. N addition was widely found to enhance soil phosphatase activities, and thereby release phosphate from organic matter to alleviate the P deficit^2^. However, the released P is rapidly absorbed by roots and microorganisms and is insufficient to balance the decrease in soil available P^4^. In addition, elevated tree growth with N addition can reduce soil available P concentration by increasing the P uptake by trees. In the present study, the amount of P absorbed by trees was not necessarily elevated by N addition, as the foliar P concentration obviously reduced with N addition (Fig. 1). Our results reflect that fertilization and other human disturbances can increase the availability of one nutrient element and simultaneously decrease the availability of other nutrient elements, which makes the responses of plants to soil nutrient availability confounding.

In the present study, SLA declined and LDMC increased with N addition, suggesting that leaf photosynthetic capacity could decline with N addition, although the growth of needles was promoted by N addition, as indicated by the higher individual leaf dry weight in the N addition treatment. Currently, the effects of soil nutrient availability on the SLA of trees are contrasting. Some studies observed an increased SLA in response to improved nutrition^23, 24, 38^, and the increased SLA was often explained by lower light availability in the canopy of the more fertile stands^35^. The decreased SLA and increased LDMC with fertilization shown in the present study were consistent with other previous studies^25, 27, 39^, while the underlying mechanisms are largely unknown. In the present study, the constraint of leaf photosynthetic capacity by P deficiency resulting from N addition is a possible explanation for the decreased SLA.

Correlation analyses showed a stronger correlation between foliar P and soil available P than that between foliar N and soil inorganic N. Moreover, SLA and LDMC correlated more strongly with soil Olsen-P than with soil inorganic N (Table 2, Fig. 3). These results are consistent with our third expectation, and demonstrate that SLA and LDMC are more strongly controlled by soil P availability than by N availability. The soil at our study site is a poor sandy soil with very low N and P availability. N addition significantly increased soil inorganic N concentration but simultaneously reduced available P concentration, which consequently increased foliar N and reduced foliar P concentrations (Fig. 2). Therefore, P limitation was aggravated by N addition and understory removal in the Mongolian pine plantation. Restriction of photosynthetic capacity by P, rather than by N, was also observed in previous studies on *Pinus sylvestris* at nutrient-poor sites^29, 39^. Both N and P play important roles in photosynthesis. However, the initial requirement of P for plant growth may be larger than that of N, because a larger relative part of foliar P, than of N, is in the nucleic acids that are required for the synthesis of proteins. Moreover, N-use efficiency is dependent on P availability, since proteins include most of the leaf N^40^.

SLA and LDMC are key traits indicating plant nutrient use strategy^17^. High SLA and low LDMC represents rapid nutrient acquisition and high biomass production, which is advantageous for plant growth in nutrient-rich environments, while low SLA and high LDMC represents the efficient conservation of nutrients and thus are advantageous for plant growth in nutrient-poor habitats^17, 40^. The reduction in SLA and the increase in LDMC often imply the deterioration of environmental conditions, as plants shift their growth strategy towards a more conservative way^41^. Therefore, our results reflect that N addition and understory removal have adverse effects on the growth of the Mongolian pine, despite the fact that they can improve short-term tree growth. Under increasing atmospheric N deposition, measures should be taken to keep the balance between N and P supply and thus maintain the sustainable development of plantations, particularly those established on soils with low P availability. The balance between soil N and P supply can be achieved by increasing P availability through fertilization, or allevating the effects of N addition on P availability through measures that regulate soil pH, such as liming. Understory removal also negatively affected the leaf growth, reflecting the positive role of understory vegetation in maintaining long-term nutrient supply, and thus understory vegetation is suggested to be preserved in forest plantations on nutrient-poor soils.

Our results demonstrate that LDMC was more responsive to variations in soil nutrient availability than SLA, as suggested by the much stronger correlation of soil Olsen-P with LDMC than with SLA (Fig. 3). These results were consistent with previous studies, which demonstrated that LDMC was more easily and accurately measured, less variable between replicates, and less dependent on leaf thickness than SLA^17, 30, 42^. Large between-replicate variation in SLA was also observed in the present study, which can be caused by variation in leaf thickness, and even errors in the measurement of leaf area or volume^17^. As a component of SLA, leaf thickness primarily varies with light intensity instead of soil fertility^31^. Enhanced nutrient supply generally results in greater within-canopy shading because of increased foliar growth, and thus causes the reduction in leaf thickness^39, 43^. This source of variation in leaf thickness could be an important interference to the actural response of SLA to soil fertility^17, 31^. LDMC defines leaf construction cost and reflects the investment of dry matter for the expansion of leaf area. Thus, LDMC theoretically plays a central role in nutrient economy by determining the rate of biomass growth and turnover, which was supported by the observations that low LDMC was associated with high growth rate and short leaf life span^16, 20^. So, our results support that LDMC is a better predictor of plant nutrient economy and growth rate in response to variations in nutrient availability than SLA.

Needle age not only had greater direct effects on the values of leaf traits than the treatment effect but also influenced the sensitivity of the of leaf traits to variations in soil nutrient availability, especially for SLA and foliar N concentration in the present study (Fig. 2). Previous year’s needles (in particular 2-year-old needles) were generally more sensitive to variations in soil nutrient availability than current-year needles, which was in agreement with our expectation. The nutrient reallocation among different-aged leaves is an important way in which plants adapt to varying environments^44^. The decline in SLA and increase in LDMC with increasing needle age explained why the young needles had higher photosynthetic rates and were more productive than the old needles^45, 46^. Under conditions of nutrient shortage, leaf photosynthesis depends on the supply of the nutrients^47^. To maintain a favorable N:P ratio and metabolic activity of new leaves, and thus sustain new foliage production, nutrients were transferred from old leaves or other tissues to new leaves^32, 48^. This internal nutrient transfer was also supported by our results that N and P concentrations declined with increasing needle age. Therefore, the internal transfer of nutrients among different-aged needles made the SLA and N concentrations of current-year needles of Mongolian pine relatively stable against the variations in nutrient supply. Our results suggest that the previous year’s needles should be taken into account when investigating leaf responses to variations in soil fertility, as they are more sensitive to variations in soil nutrient availability and account for a large proportion of total foliage biomass.

In summary, this study found that five years of N addition significantly elevated LDMC and reduced SLA in a young Mongolian pine plantation, which can be largely ascribed to the intensified P deficiency resulting from the N addition. Understory removal had weaker effects than N addition. Given that SLA and LDMC are key traits indicating plant nutrient economy and growth rate, our results suggest that N addition and understory removal can have adverse effects on the growth of Mongolian pine established on soils with low available N and P. Thus, in order to maintain the sustainable development of forest plantations under increasing atmospheric N deposition, measures are required to keep the balance between N and P supplies. Additionally, LDMC correlated more closely with soil available P than SLA, suggesting that it is a better predicator of variations in nutrient availability than SLA. The previous year’s needles were more sensitive to variations in nutrient availability than the current-year needles for nutrient-dependent leaf traits, and thus should be taken into account when investigating leaf responses to variations in soil fertility.

## Materials and Methods

### Site description and experimental design

The study site is located at the Daqinggou Ecological Station, Institute of Applied Ecology, Chinese Academy of Sciences (42°58′N, 122°21′E, 260 m asl). The site is located in a semiarid region, and has a temperate climate. The highest and lowest average monthly temperatures were 23.8 °C in July and −12.5 °C in January, and the average annual temperature at the site was 6.4 °C. The average annual precipitation was 450 mm, with over 60% of the total precipitation occurring from June to August. The soil is a nutrient-poor sandy soil developed from eolian deposit (Typic Ustipsamment), with 90.9% sand, 5.0% silt, and 4.1% clay.

In April 2011, we selected a 13-year-old pure Mongolian pine plantation for the manipulative experiment. Mongolian pine was the main afforestation tree species in northern China in past decades for soil conservation purpose. The plantation was established with 2 m × 5 m spacing on degraded grassland with flat topography. The canopy closure was 60%, the average tree height was 3.9 m, and the stem diameter at breast height was 7.0 cm at the onset of the experiment. The understory vegetation had approximately 80% cover, and mainly consisted of *Artemisia scoparia, Cannabis sativa, Setaria viridis, Chenopodium acuminatum*, and *Lespedeza daurica*. The aboveground biomass of understory vegetation was approximately 337 g m^−2^.

We designed a randomized block experiment with four blocks. Each block consisted of four treatments in a 2 × 2 factorial combination of N addition (with and without N addition) and understory removal (with or without understory removal). Four treatments including control (the control without any disturbance), N+ (addition of 10 g N m^−2^ year^−1^), U− (removal of all understory vegetation), and N + U− (combination of N+ and U−). We established four blocks in the 2 ha Mongolian pine plantation, and each block consisted of four 20 m × 30 m plots with at least a 5 m buffer zone between adjacent plots. The four treatments were randomly arranged in the four plots of each block. For the N addition, urea was dissolved in water and spread monthly in the N+ and N + U− plots during the growing season (from May to September). 2 g N m^−2^ was spread every time, with a total of 10 g m^−2^ year^−1^. An equal amount of water was spread in the control and U− plots. For the understory removal, at the beginning of the experiment, a 50% (w/v) acetochlor solution was spread evenly in U− and N + U− plots to kill the understory vegetation. Afterward, the remaining and recolonizing understory vegetation was removed by hand monthly during the growing season. The acetochlor is easily degraded, and it has minimal impacts on soil ecosystems^49^.

### Foliage sampling and measurements

In August 2016, three trees were randomly chosen in each plot to collect needles. To avoid excessive defoliation and effects of crown position, eight branches were selected in different directions in the middle layer of the tree crown for each sampling tree. All the needles were classified by age class (including the current-year; 1-year-old; 2-year-old; and 3-year-old) and approximately 200 bunches of fully expanded, fresh needles for each needle age in each plot were homogenized by plot. Thirty bunches of fresh needles were selected to determine the leaf dry matter content (LDMC, mg·g^−1^), specific leaf area (SLA, cm^2^·g^−1^) and individual needle dry weight (mg). First, we measured the length of each fresh needle with a vernier caliper, and then measured the total volume of the 30 bunches of needles using the water displacement method^50^. Then, the needles were placed in distilled water for 12 h in the dark prior to the measurement of turgid leaf weight. Finally, we measured the dry weight of the needles by oven-drying the needles at 65 °C to a constant weight. The SLA was calculated as the ratio of leaf area to leaf dry weight, and leaf area was calculated using the following equation^50^:

$$LA=2L(1+\pi /{\rm{n}})\sqrt{nV/\pi L}$$

where *LA* is leaf area; *L* is the average length of needles; π = 3.14; *n* is the number of needles per bunch (the value is 2 for Mongolian pine); and *V* is the volume of needles. All the oven-dried needles were then ground for nutrient analysis. Total N and P concentrations in the needles were determined using a continuous-flow autoanalyzer (AutoAnalyzer III, Bran + Luebbe GmbH, Germany) after digestion in 5 ml H_2_SO_4_ with a catalyst (mixture of CuSO_4_ and K_2_SO_4_)^51^. The crown average value of each leaf trait was calculated as the sum of the value of each age class multiplied by its mass proportion in the total leaf biomass.

### Soil sampling and measurements

At the same time as foliage sampling, surface mineral soils (0–10 cm) were collected using a soil corer with an inner diameter of 2.5 cm. 30 soil cores were collected from each plot and homogenized into one sample. Soil samples were sieved through 2 mm mesh and divided into two subsamples: one subsample was air-dried for the determination of soil pH, soil organic C, total N, total P and Olsen-P; the second subsample was stored at 4 °C for less than 5 days until the measurement of soil water content, microbial biomass C, NO_3_-N and NH_4_^+^-N.

Soil water content was measured from mass loss after drying at 105 °C to a constant weight. Soil pH was measured with a pH meter in a 1:2.5 soil/water suspension^51^. Soil organic C was determined by the H_2_SO_4_–K_2_Cr_2_O_7_ oxidation method^52^. Soil total N and P concentrations were determined using a continuous-flow autoanalyzer (AutoAnalyzer III, Bran + Luebbe GmbH, Germany) after digestion in 5 ml H_2_SO_4_ with a catalyst (mixture of CuSO_4_ and K_2_SO_4_)^51^. Soil Olsen-P concentration was analyzed colorimetrically using the molybdate blue method after the soil was extracted with 0.5 mol L^−1^ NaHCO_3_ at pH = 8.5^53^. Concentrations of soil NO_3_-N and NH_4_^+^-N were analyzed colorimetrically on the autoanalyzer after the soil was extracted with 2 M KCl solution. Soil inorganic N was calculated as the sum of NO_3_-N and NH_4_^+^-N. Microbial biomass C was determined by the fumigation extraction method^54^.

### Statistical analysis

To test the effects of N addition (N), understory removal (U) and their interactions (U × N) on the soil properties and crown average values of leaf traits, we performed the analysis of variance (ANOVA) using a general linear model with N and U as fixed factors, and the block as a random factor. In addition, post hoc multiple comparisons of means were used to compare differences among all the four treatments using the least significant difference (LSD) test. All data were tested for homogeneity of variance before performing ANOVA. We examined the relationship among soil properties by Pearson’s correlation analysis and principal component analysis (PCA). Soil data were ln (*x* + 1) transformed during PCA analysis. Relations between needle traits and soil nutrient availability (as indicated by soil inorganic N and Olsen-P), were also examined using the Pearson’s correlation analysis. ANOVA and correlation analyses were conducted with SPSS software (16th edition, Chicago, USA), and PCA was performed on CANOCO 4.5 software. Differences obtained at *p* < 0.05 were considered significant.

## Electronic supplementary material

Supplement Table 1 and 2
