# Supplementary material for: Altered leaf functional traits by nitrogen addition in a nutrient-poor pine plantation: A consequence of decreased phosphorus availability
Source: Sci Rep. 2017 Aug 7;7:7415. doi: 10.1038/s41598-017-07170-3 (PMC5547060; doi:10.1038/s41598-017-07170-3)
Supplement: Supplementary file 1 — Supplement Table 1 and 2 [file 41598_2017_7170_MOESM1_ESM.doc]

**Altered leaf functional traits by nitrogen addition in a nutrient-poor pine plantation: A consequence of decreased phosphorus availability**

Lin-Lin Zheng^1,2,3^, Qiong Zhao^1,2*^, Zhan-Yuan Yu^1,2^, Shan-Yu Zhao^1,2,3^, De-Hui Zeng^1,2^

*^1^Key Laboratory of Forest Ecology and Management, Institute of Applied Ecology, Chinese Academy of Sciences, Shenyang 110016, PR China*

^2^ *Daqinggou Ecological Station, Institute of Applied Ecology, Chinese Academy of Sciences, Shenyang 110016, PR China*

*^3^University of Chinese Academy of Sciences, Beijing 100049, PR China*

**Supplementary Table 1** Results from two-way ANOVA (*F* and *p* values) of the main effects of understory removal (U) and N addition (N) and their interactions (U × N) on soil properties and the average values of needle traits at the crown level. Significant *F* and *p* values are highlighted in bold.

| **Variable** |  | **U** | **N** | **U × N** |
| --- | --- | --- | --- | --- |
| **pH** | *F* | 2.069 | **25.870** | 0.012 |
|  | *p* | 0.178 | **< 0.001** | 0.914 |
| **Water content** | *F* | 1.218 | 0.665 | 0.133 |
|  | *p* | 0.293 | 0.432 | 0.723 |
| **NO_3_^-^-N** | *F* | **6.794** | **140.816** | **7.848** |
|  | *p* | **0.024** | **< 0.001** | **0.017** |
| **NH_4_^+^-N** | *F* | **63.067** | **407.296** | **56.307** |
|  | *p* | **< 0.001** | **< 0.001** | **< 0.001** |
| **Olsen-P** | *F* | 0.411 | **36.593** | **9.333** |
|  | *p* | 0.535 | **< 0.001** | **0.011** |
| **MBC** | *F* | **11.499** | **4.828** | 2.503 |
|  | *p* | **0.006** | **0.050** | 0.142 |
| **SOC** | *F* | 0.671 | 3.299 | 0.364 |
|  | *p* | 0.430 | 0.097 | 0.559 |
| **Total P** | *F* | 2.456 | 0.168 | 0.022 |
|  | *p* | 0.145 | 0.690 | 0.885 |
| **Total N** | *F* | 3.595 | **12.197** | 0.015 |
|  | *p* | 0.085 | **0.005** | 0.904 |
| **DW** | *F* | 0.953 | **14.655** | 0.035 |
|  | *p* | 0.350 | **0.003** | 0.855 |
| **SLA** | *F* | 1.028 | **4.649** | 1.912 |
|  | *p* | 0.332 | **0.054** | 0.194 |
| **LDMC** | *F* | 0.630 | **6.050** | **8.023** |
|  | *p* | 0.444 | **0.032** | **0.016** |
| **LNC** | *F* | 1.463 | **10.857** | 0.643 |
|  | *p* | 0.252 | **0.007** | 0.440 |
| **LPC** | *F* | 0.240 | **41.666** | 0.007 |
|  | *p* | 0.634 | **< 0.001** | 0.935 |
| **N: P ratio** | *F* | 0.268 | **66.182** | 0.106 |
|  | *p* | 0.615 | **< 0.001** | 0.750 |

*MBC* soil microbial biomass C, *SOC* soil organic C, *DW* individual needle dry weight, *SLA* specific leaf area, *LDMC* leaf dry matter content, *LNC* leaf N concentration, *LPC* leaf P concentration.

**Supplementary Table 2** Average values (and standard deviations) of several parameters in relation to needle age class.

| **Variable** | **Age class** | **Average** | **Standard error** | **Min** | **Max** |
| --- | --- | --- | --- | --- | --- |
| **DW**  **(mg)** | Current-year | 32.31 **c** | 1.78 | 23.80 | 46.22 |
|  | 1-year | 44.21 **b** | 1.27 | 36.76 | 54.78 |
|  | 2-year | 74.84 **a** | 2.79 | 55.57 | 89.55 |
|  | 3-year | 76.31 **a** | 2.65 | 56.32 | 95.23 |
| **SLA**  **(cm^2^ g****^-1^)** | Current-year | 104.45 **a** | 2.16 | 89.64 | 118.06 |
|  | 1-year | 97.51 **b** | 1.72 | 86.80 | 112.39 |
|  | 2-year | 75.53 **c** | 1.71 | 65.46 | 90.72 |
|  | 3-year | 69.02 **d** | 1.58 | 58.58 | 78.64 |
| **LPC**  **(mg g^-1^)** | Current-year | 1.82 **a** | 0.06 | 1.47 | 2.38 |
|  | 1-year | 1.29 **b** | 0.04 | 1.02 | 1.56 |
|  | 2-year | 1.03 **c** | 0.03 | 0.84 | 1.23 |
|  | 3-year | 0.90 **d** | 0.02 | 0.79 | 1.12 |
| **LNC**  **(mg g^-1^)** | Current-year | 21.05 **a** | 0.27 | 18.42 | 23.17 |
|  | 1-year | 19.91 **b** | 0.33 | 17.66 | 22.53 |
|  | 2-year | 17.37 **c** | 0.36 | 15.17 | 19.69 |
|  | 3-year | 14.35 **d** | 0.35 | 12.48 | 17.30 |
| **LDMC**  **(mg g^-1^)** | Current-year | 364.38 **d** | 5.99 | 328.71 | 405.30 |
|  | 1-year | 393.26 **c** | 6.02 | 357.98 | 435.11 |
|  | 2-year | 421.02 **b** | 4.37 | 392.72 | 451.45 |
|  | 3-year | 438.60 **a** | 4.81 | 408.87 | 464.24 |
| **N: P** | Current-year | 11.74 **b** | 0.41 | 8.34 | 14.97 |
|  | 1-year | 15.69 **a** | 0.58 | 11.46 | 19.27 |
|  | 2-year | 17.18 **a** | 0.76 | 12.34 | 22.36 |
|  | 3-year | 16.13 **a** | 0.55 | 12.94 | 19.25 |

*DW* individual needle dry weight, *SLA* specific leaf area, *LDMC* leaf dry matter content, *LNC* leaf N concentration, *LPC* leaf P concentration.
